# Grayanotoxin Poisoning: ‘Mad Honey Disease’ and Beyond

**DOI:** 10.1007/s12012-012-9162-2

**Published:** 2012-04-19

**Authors:** Suze A. Jansen, Iris Kleerekooper, Zonne L. M. Hofman, Isabelle F. P. M. Kappen, Anna Stary-Weinzinger, Marcel A. G. van der Heyden

**Affiliations:** 1 Honours Program CRU2006 Bachelor, University Medical Center Utrecht, Heidelberglaan 100, 3584 CX Utrecht, The Netherlands; 2 Department of Pharmacology and Toxicology, University of Vienna, Althanstrasse 14, 1090 Vienna, Austria; 3 Division of Heart and Lungs, Department of Medical Physiology, University Medical Center Utrecht, Yalelaan 50, 3584 CM Utrecht, The Netherlands

**Keywords:** Grayanotoxin, Conduction block, Arrhythmia, Hypotension, Mad honey, *Rhododendron*, Complementary medicine, *Ericaceae*, Geography

## Abstract

Many plants of the *Ericaceae* family*, Rhododendron*, *Pieris, Agarista* and *Kalmia,* contain diterpene grayanotoxins. Consumption of grayanotoxin containing leaves, flowers or secondary products as honey may result in intoxication specifically characterized by dizziness, hypotension and atrial-ventricular block. Symptoms are caused by an inability to inactivate neural sodium ion channels resulting in continuous increased vagal tone. Grayanotoxin containing products are currently sold online, which may pose an increasing risk. In humans, intoxication is rarely lethal, in contrast to cattle and pet poisoning cases. Scientific evidence for the medicinal properties of grayanotoxin containing preparations, such as honey or herbal preparation in use in folk medicine, is scarce, and such use may even be harmful.

## Introduction

Plants contain numerous compounds that, when beneficial to humans, are categorized as “medicinal” and when harmful they are termed “poisonous”. Secondary products derived from plants, such as honey, can contain a number of chemical compounds that, depending on their concentration and application, can also be considered medicinal or poisonous [1]. Grayanotoxin containing honey, called “mad honey”, can cause dramatic effects when ingested as has already been recorded by the Greek warrior-writer Xenophon in 401 BC in his Anabasis “… but the swarms of bees in the neighborhood were numerous, and the soldiers who ate of the honey all went of their heads, and suffered from vomiting and diarrhea, and not one of them could stand up, but those who had eaten a little were like people exceedingly drunk, while those who had eaten a great deal seemed like crazy, or even, in some cases, dying men. So they lay there in great numbers as though the army had suffered a defeat, and great despondency prevailed. On the next day, however, no one had died, and at approximately the same hour as they had eaten the honey they began to come to their senses; and on the third or fourth day they got up, as if from a drugging” [2]. More recently, grayanotoxin containing honey has been described as the cause of symptoms in a large number of near fatal casualties. This may pose an unexpected health risk, as honey and plant preparations containing grayanotoxin are currently sold online. Here, we provide mechanistic insights into the mode of action of grayanotoxins, review mad honey intoxications from the Black Sea region and other locations, and review poisoning caused by ingestion of parts of grayanotoxin containing plants. Finally, we discuss studies demonstrating the potential medicinal advantages of grayanotoxins that, although to a limited extent, provide a scientific basis for centuries old claims of health advantages of mad honey and grayanotoxin containing preparations.

## Grayanotoxins

### Origin and Chemical Structure

**Fig. 1:**
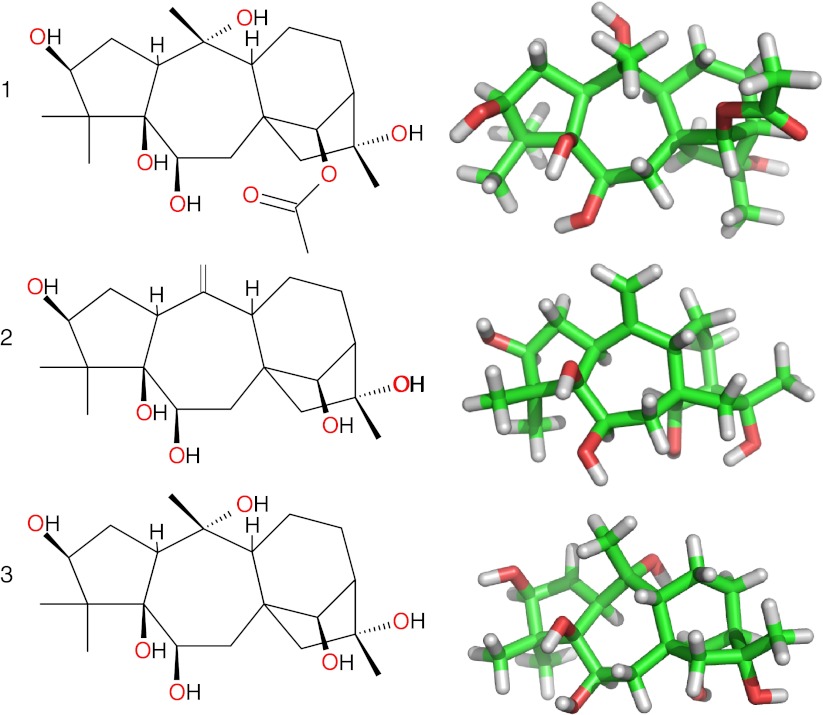
Structure formulas (*left panel*) and 3-D representations (*right panel*) of grayanotoxins 1, 2 and 3. *Color code* *gray* hydrogen atoms, *red* oxygen atoms, *green* carbon atoms

Grayanotoxins, also known as andromedotoxin, acetylandromedol or rhodotoxin, can be derived from the leaves, twigs or flowers of plants belonging to genera of the *Ericaceae* (heath) family [3–7], comprising among others the *Rhododendron*, *Pieris, Agarista* and *Kalmia* genera. The toxin is also present in a number of products originating from the family members, such as honey, labrador tea, cigarettes and a variety of decoctions used in alternative medicine [5, 8]. Its chemical structure has been fully elucidated as a diterpene, a polyhydroxylated cyclic hydrocarbon with a 5/7/6/5 ring structure that does not contain nitrogen [9]. More than 25 grayanotoxin isoforms have been isolated from *Rhododendron* [10]. Three members of the large grayanotoxin family (Fig. 1) have been demonstrated to be of particular relevance in the reported clinical cases [11]. Grayanotoxin 1 and 2 have been found in the honey, leaves and flowers of *Rhododendron ponticum* and *Rhododendron flavum* as reported in multiple case reports in the eastern Black Sea area [3, 5, 12]. Grayanotoxin 1 present in *Rhododendron simsii*, has been reported from a case in Hong Kong [8] while in the honey from Grouse Mountain, BC and Canada, which causes a similar type of poisoning, only grayanotoxins 2 and 3 were found [12]. Currently, grayanotoxin 1 and 3 are thought to be the principal toxic isomers [4, 11–13].

### Molecular Interference of Grayanotoxin on Sodium Channel

The toxicity of grayanotoxin lies in its ability to bind to the group II receptor site in voltage-gated sodium channels (Na_v_1·*x*) within the cell. In general, the sodium channel alpha subunit protein consists of 24 interconnected membrane spanning alpha-helixes that are organized as four repeats of 6 alpha-helixes each (S1-S6). In the resulting channel, the four S6 domains align the pore region. Tests with a hydrophilic grayanotoxin analog, desacyl asebotoxin VII, utilizing a squid axonal membrane, demonstrated that the binding site for granayotoxin most probably resides on the internal surface of the membrane as this hydrophilic analog was found to be active only when applied from the cytoplasmic side [14]. In a mutation analysis of Na_v_1.4, Maejima et al. [15] demonstrated an important contribution of all four S6 domains in grayanotoxin interaction. Grayanotoxin binding modifies the channels configuration to such an extent that it prevents sodium channel inactivation, rendering the cell in a depolarized, and thus, activated state. Grayanotoxins bind to the channel only in its open state, and hereafter, the activation potential of the modified sodium channel is shifted in the direction of hyperpolarization [15]. Whether grayanotoxins interact with other members of the sodium channel family remains to be elucidated.

### Effects of Grayanotoxin on Nervous System

The voltage-gated sodium channels of the neurons are most likely a prominent target of grayanotoxins. In animal studies, Onat et al. [16] found that injecting a small dose equivalent to 50 mg of honey intracerebroventricularly in anaesthetized albino rats caused marked bradycardia and respiratory depression. However, a much larger amount of extract, equivalent to 1 and 5 g/kg, was needed intraperitoneally to obtain the same results [16]. This might indicate the important role of the central nervous system in grayanotoxin pathophysiology compared to the peripheral nervous system. Alternatively, intraperitoneal injection may result in increased elimination rates. Nonetheless, in bilaterally vagotomized rats, bradycardia did not occur after the administration of grayanotoxin [16], showing that peripheral vagal stimulation appears to play a role in grayanotoxin-induced bradycardia. As the muscarinic receptors that mediate vagal stimulation in the myocardium are of the M_2_-subtype, the effects of atropine, a non-specific anti-muscarinic agent and AF-DX 16, a selective M_2_-receptor antagonist, were analyzed in another rat study [5]. Rats were injected intraperitoneally with extract equivalent to 1 or 5 g/kg of honey. When marked bradycardia, defined as approximately 75 % of the control value, occurred, either 2 mg/kg atropine sulfate or 20 mg/kg AF-DX 16 was administered intraperitoneally. It was observed that atropine abrogated both the grayanotoxin-induced bradycardia and respiratory depression, while AF-DX 16 only counteracted the bradycardic effect. From these results, it was concluded that M_2_-receptor subtypes are involved in grayanotoxin-induced cardiotoxicity, but not in respiratory toxicity [5].

## Mad Honey Disease, Clinical Features and Geographical Distribution

### Honey Containing Grayanotoxin

The best studied and most numerous cases of grayanotoxin toxicity known thus far are those classified as mad honey disease. Patients have normally ingested variable amounts (20–200 g) of grayanotoxin containing honey [17–22]. Contamination of the honey occurs mainly in the eastern Black Sea region of Turkey, where bees produce honey from nectar derived from the *Rhododrendron ponticum* and *Rhododendron luteum*. Since most of the local beekeepers produce honey at a small scale, the final products can be obtained from a small area or even a single bee hive and contain a considerable concentration of grayanotoxin. Grayanotoxin concentrations were reported in samples of North-American honey; White and Riethof [23] reported 100 ppm in a sample of NC honey, most likely produced from mountain laurel nectar (*Kalmia latifolia*). In a honey sample from Grouse Mountain, British Columbia, Canada, Scott et al. [13] reported 3 and 7 ppm for grayanotoxin 2 and 3 respectively. In regions where production has been scaled up, the final product most often consists of a mixture of honey produced at different locations, thereby limiting the chance for severe grayanotoxin contamination by dilution. However, some beekeepers produce mad honey purposely for its supposed therapeutic effects.

### Clinical Characteristics

A number of clinical signs have been associated with mad honey disease [3, 4]. Most often hypotension, cardiac rhythm disorders (first, second and third degree AV block, asystole, and sinus bradycardia), nausea and/or vomiting, sweating, dizziness and impaired consciousness have been observed. More rarely, syncope, blurred vision or diplopia and salivation have been described. In some rare cases, convulsion [24], atrial fibrillation [25], asystole and myocardial infarction were observed [19, 26, 27]. Symptoms occur most often within 20 min to 3 h after ingestion and remain for 1–2 days. In severe cases presented at the emergency room, treatment most often consists of atropine (0.5 mg once, or twice in case of non-response with respect to blood pressure and heart rate; 2 mg once) and saline (100 mL/h) [19, 27]. The main symptoms are believed to be caused by continued sodium channel activation, cell depolarisation and hence stimulation of the vagal nervous system. Whether grayanotoxin also acts directly on cardiomyocytes via sodium channel interference still has to be determined. While a number of cases of mad honey ingestion are believed to be accidental, deliberate intoxication may originate from the general belief that mad honey can act as an aphrodisiac, or as a treatment for gastritis and peptic ulcers, weakness, arthritis, diabetes or hypertension [28, 29]. It is speculated that such therapeutic intake may form the basis of chronic mad honey intoxication syndrome that is characterized by sinus bradycardia, first and second degree AV block, dizziness and presyncope [21].

### Geographical Distribution of Mad Honey Disease

Most known cases of mad honey disease are restricted to the Black Sea region [4]. However, transport and export of locally produced honey from Turkey or elsewhere, and geographical spreading of the Turkish population, may result in cases in other regions. For example, some cases have been reported from other parts of Turkey [30], Germany [31, 32], Austria [33, 34], Switzerland [35] and Korea [20, 36, 37]. The latter country imported considerable amounts of toxic honey, which has been prohibited since 2005 [20]. Furthermore, some old and recent cases of local wild honey poisoning from Nepal [17, 38, 39] and Reunion Island [40] underline the occurrence of grayanotoxin intoxication by honey produced outside of Turkey.

## Grayanotoxin Poisoning Unrelated to Mad Honey

### Intoxication of Humans

Although mad honey is the most common cause of grayanotoxin intoxication, it is not the only one. The ingestion of leaves, nectar and flowers of plants containing grayanotoxin can cause intoxication too. These cases (Table 1) are very rare and occur mainly, but not exclusively, in Asia. The intake can arise from false beliefs in medicinal properties of the plant. In Hong Kong, a 57-day-old baby suffered from grayanotoxin intoxication, presenting respiratory distress, bradycardia, hypotension, constricted pupils, salivation and muscle twitching, after the grandmother added a decoction of *Rhododendron simsii* to the bottle of milk, convinced it would be beneficial to the airways [8]. Kim et al. [41] reported grayanotoxin intoxication in three patients who ingested either blossom leaves or medicinal preparations made from *Rhododendron* species. A 61-year-old female presented conduction disturbances and junctional escape beats while two males of 70 and 73 years of age respectively showed sinus bradycardia. All responded well to atropine [41]. A 59-year-old man from Korea drank an herbal tea made from *Rhododendron brachycarpum* and presented signs of grayanotoxin poisoning (bradycardia, hypotension, dizziness, sweating) [42].

**Table 1:** Overview of grayanotoxin intoxication cases in humans, unrelated to mad honey requiring hospitalization

Age (years)	Sex	Origin	Cause	Purpose	Clinical signs	Treatment	Clinical course	References
0	M	Hong Kong	Milk containing *R. simssii*	Relieve airway problems	Respiratory distress, sinus bradycardia (90–100 bpm), hypotension (62/33 mmHg), constricted non-reactive pupils, salivation, muscle twitching	Mechanical ventilation Benzodiazepine i.v. Atropine (0.1 mg)	Fast improvement after atropine hospital discharge at day 8 without complications	[8]
71	M	South Korea	Ate *Rhododendron* flowers	Unknown	Vomiting, visual disturbance, sinus bradycardia (47 bpm) hypotension (90/60 mmHg)	Atropine (0.5 mg) Saline i.v. (1,000 ml)	Hospital discharge at day 2 without complications	[41]
70	M	South Korea	Drank *Azalea* wine	Unknown	Dizziness, chest discomfort, sinus bradycardia (37 bpm), hypotension (70/50 mmHg)	Atropine (0.5 mg) Saline i.v.	Hospital discharge at day 3 without complications	[41]
61	F	South Korea	Drank *Rhododendron* flower juice	Unknown	Dizziness, general weakness, chest discomfort, bradycardia (40 bpm), junctional escape beat	Atropine (0.5 mg) Saline i.v.	Hospital discharge at day 2 without complications	[41]
59	M	South Korea	Drank boiled *R.* *brachycarpum* extract	Anti-hypertensive	Dizziness, weakness, chest discomfort, cold sweats, sinus bradycardia (36 bpm), atrio-ventricular dissociation, hypotension (68/38 mmHg)	Atropine (1.0 mg) Saline i.v.	Fast but temporary improvement of bradycardia hospital discharge at day 3 with 1st° AV block	[42]
9	M	South Korea	Ate 10 *R. sclippenbachii* flowers	Unknown	Impaired consciousness, delirium	Saline i.v.	Symptoms resolved in 17 h	[43]
1	F	USA	Eating garden *P. japonica*	Unknown	Vomiting, pallor, bradycardia (40 bpm)	Atropine, charcoal, mechanical ventilation	Improved fully	[44]
76	M	USA	Drank tea made from *P. japonica*	Unknown	Vomiting, blurred vision, seizure, bradycardia, hypotension (70 mmHg)	Unknown	Improved fully	[44]
76	M	South Korea	Ingested 10 *Azalea* blossoms	Unknown	Dizziness, general inertia, sinus bradycartia (45 bpm), hypotension (70/50 mmHg), drowsiness	Atropine (1.0 mg)	Fully recovered, hospital discharge after 1 day	[45]
58	M	South Korea	Ingested 50 *R. sclippenbashii* blossoms	To relieve thirst	Hypotension (80/40 mmHg), bradycardia (30 bpm)	Atropine (0.5 mg) Saline i.v.	Still symptoms at 24 h, recovered later	[46]
28	F	Reunion Island	Drank tea made from *Agauria salicifolia*	Unintended	Vomiting, diarrhea, facial flush, sweating, fatigue, hypotension (60/40 mmHg), sinus bradycardia (40 bpm)	Saline i.v.	Fully recovered, hospital discharge after 1 day	[47]

Children that eat the plants are also at risk. A 9-year-old boy from Korea consumed about ten *Rhododendron schlippenbachii* flowers and, besides the common signs of grayanotoxin intoxication, presented with impaired consciousness and delirium 26 h later. All symptoms resolved completely after a further 17 h [43]. In the USA, ingestion of *Ericaceae* by children has occurred as well. A 21-month-old girl was admitted to the hospital after eating a *Pieris japonica* garden plant [44]. She presented with bradycardia (40 bpm), pallor and vomiting, and following treatment with atropine her condition improved [44].

In adults, a lack of knowledge or a (mis)calculated risk can be a reason for intoxication. Some Koreans believe that the *Azalea* (*Rhododendron mucronulatum*) is non-poisonous, even though the toxicity of *Rhododendron* species is commonly understood. Blossoms of the *Azalea* are therefore ingested in different forms, enabling intoxication. A 76-year-old man was hospitalized when presenting grayanotoxin intoxication (hypotension, sinus bradycardia, dizziness, general inertia) after ingesting the blossom of this plant [45]. A patient in Korea consumed 50 blossoms of the *R. schlippenbachii* to get rid of his thirst, while aware of the poisonous properties [46].

A 28-year-old woman from Saint-Paul-de-la-Reunion, one of the Mascarene Islands close to Madagascar in the Indian Ocean, who mistakenly drank an herbal tea from leaves of the *Agauria salicifolia,* a plant from the *Ericaceae* family, presented the typical signs of grayanotoxin intoxication [47]. The same happened in the USA to a 76-year-old man that presented bradycardia, hypotension, vomiting, blurred vision and seizure, after drinking tea made from *P. japonica* [44].

Finally, misidentification of plant species may cause intoxication. The toxic sheep laurel (*Kalmia angustifolia*) resembles *R. tomemtosum* in appearance [48]. *R. tomentosum* and *R. groenlandicum*, named Northern and Bog Labrador Tea, are used for preparing herbal teas for their medicinal qualities according to Native Americans. The Labrador Tea itself is said to be mildly toxic and should be drunk in small quantities only.

### Intoxication in Cattle and Pets

In humans, there are few cases known of grayanotoxin intoxication unrelated to mad honey. Mainly cattle are susceptible to grayanotoxin intoxication, but it has also been reported in sheep, goats and donkeys. Plants known for animal intoxication are the *Rhododendron*, the laurel (*Kalmia*) and the Japanese *Pieris*. *Rhododendron* intoxication is most common in winter and early spring, since *Rhododendron* leaves are perennial [49]. *Rhododendron* has a toxic dose of 0.2 % body weight in cattle, while *Kalmia* has a toxic dose of 0.4 % body weight [49]. When an animal presents gastrointestinal tract irritation, cardiac arrhythmias, and neurological symptoms, grayanotoxin intoxication should be considered. The symptoms of grayanotoxin intoxication in cattle most commonly present themselves 3–14 h after ingestion and remain present for 2 days. The animal may recover, but in contrast to most human cases, the intoxication is often lethal [50–53]. Besides cardiovascular instability, aspiration of the vomitus is a common cause of death [49]. Although cattle-keepers may be well aware of the toxic effects of grayanotoxin containing plants, still, poisoning may occur when animals are fed by bystanders. This was observed in a case of goats that ate *P. japonica* prune debris thrown into their pasture by the neighbor [54]. Nubian goats of the Riverbanks Zoological Park were given a *Rhododendron indica* branch by a zoo visitor and developed grayanotoxin poising that was subsequently confirmed by urine and feces analysis [55].

Leengoed and Amerongen described an interesting case of intoxication in a herd of 65 sheep that ingested *R. ponticum*. The intoxication followed an unexpected course. After treatment with laxatives, the condition of the sheep improved but 36 h after intoxication, the condition of almost all sheep deteriorated again. Leengoed and Amerongen hypothesized this could be due to shut down of rumination caused by intoxication. After initial recovery, rumination was reestablished instigating renewed intoxication. A rumenotomy performed 18 h after intoxication on 1 sheep proved curative [56].

Domestic animals, such as cats and dogs, can also be exposed to grayanotoxin, usually in the form of the *Azalea*. The Animal Poison Control Center (APCC), part of the American Society for the Prevention of Cruelty to Animals (ASPCA), has received 188 cases of *Azalea* ingestion of dogs and cats throughout America during January 2001 to December 2003. The signs included vomiting, depression, diarrhea and anorexia. The animals usually had access to the plants because they were accessible in the garden or brought into the house as ornamental plants [57].

## Grayanotoxin and Complementary Medicine

Mad honey is often consumed for its believed medicinal potential [21] and grayanotoxin containing herbal preparations are in use in traditional Chinese medicine. This presumed therapeutic usefulness has become plausible, now that several studies have investigated the therapeutic action of grayanotoxin. The mad honey intoxication cases demonstrate a clear effect of grayanotoxin on the heart with dramatic decrease in blood pressure and heart rate [3]. Despite this evidence of powerful anti-hypertensive action, the potential medical use of grayanotoxin itself, or new grayanotoxin analogs, in cardiovascular disease has not (yet) been fully investigated. On the other hand, the efficacy of grayanotoxin in diabetes treatment was shown in rats with streptozotocin-induced diabetes mellitus. Three days after streptozotocin treatment, blood glucose levels were measured just before and 1 h after administering grayanotoxin containing honey (50 mg/kg orally). Both in the control group as well as in the streptozotocin-treated group, mean blood glucose levels decreased significantly after mad honey administration. Moreover, the lipid levels (cholesterol, triglyceride and VLDL) measured on the third day of subsequent mad honey application decreased significantly in control as well as in streptozotocin-treated rats. A possible explanation could be the grayanotoxin-mediated stimulation of the parasympatic nervous system or the M_2_-muscarinic receptors causing increased insulin secretion [58]. Species of the *Rhododendron* genus have been shown to possess many bioactive compounds [10]. Although the number of studies on biological actions, such as anti-viral or anti-bacterial, of various *Rhododendron* preparations is increasing, it is largely unknown whether grayanotoxin is the active compound. In one study, *Rhododendron ferrugineum* extract was added to the so-called Vero cells with a herpes simplex virus-1 (HSV-1) inoculum. Subsequently, HSV-1 binding and penetration was markedly decreased. The presence of grayanotoxin as an active compound, however, was ruled out [59]. Unfortunately, this study that includes analysis of the active compounds responsible for the observed effects is one of the rare exceptions.

Research on the medicinal use of the *Rhododendron* species, while still in its early stages, has revealed initial results confirming folk belief to some extent. Currently, unregistered *Rhododendron* extracts containing grayanotoxin are sold online. One example is the so-called Nao Yang Hua made from the Japanese *Azalea* (*Rhododendron molle*), which is said to benefit numerous ailments, including rheumatoid arthritis and fungal skin infection. Grayanotoxin poisoning cases raise concerns about the uncontrolled sales of potent plant extract from the *Rhododendron* genus. For now, the presence of grayanotoxin in complementary medicine should be avoided.

## Conclusions

Grayanotoxins are present in many members of the *Ericaceae* plant family. They interfere in normal sodium channel functioning, resulting in continued stimulation of the vagal nervous system. Grayanotoxin intoxication most often occurs as a result of mad honey consumption in Turkey; however, additional cases have been found worldwide due to consumption of exported Turkish or locally produced mad honey. Poisoning by consumption of *Ericaceae* leaves or flowers or derived herbal preparations is rare in humans while many lethal cases are reported in cattle and pets. Medicinal use of grayanotoxin is not well understood and care should be taken when consuming grayanotoxin containing herbal preparation.
